# Associations of mode of travel to work with physical activity, and individual, interpersonal, organisational, and environmental characteristics

**DOI:** 10.1016/j.jth.2018.01.009

**Published:** 2018-06

**Authors:** Harriet Batista Ferrer, Ashley Cooper, Suzanne Audrey

**Affiliations:** a Bristol Medical School: Population Health Sciences, University of Bristol, UK; b Centre for Exercise, Nutrition and Health Sciences, School for Policy Studies, University of Bristol, 8 Priory Road, Bristol BS8 1TZ, UK; c National Institute for Health Research Bristol Biomedical Research Centre, University Hospitals Bristol NHS Foundation Trust and University of Bristol, Education and Research Centre Level 3, Upper Maudlin Street, Bristol BS2 8AE, UK

**Keywords:** SD, Standard Deviation, UK, United Kingdom, GPS, Global Positioning System, MVPA, Moderate to Vigorous Physical Activity, CPM, Counts per minute, AccGPS, Combined Accelerometer and GPS data, GIS, Geographical Information System, SNR, Signal to Noise Ratio, OR, Odds Ratio, aOR, Adjusted Odds Ratio, CI, Confidence Interval, Physical activity, Walking, Active travel, Commute, Workplace policies

## Abstract

**Introduction:**

Encouraging walking during the daily commute is a potential strategy for increasing physical activity levels. This study aimed: (i) to examine, and compare by travel mode, the objectively measured physical activity of a working adult population, and, (ii) to identify associations between mode of travel to work and a range of individual, interpersonal, organisational and environmental characteristics.

**Methods:**

Employees (n=654) recruited from 87 workplaces in geographically distinct areas provided data through accelerometers, Global Positioning System (GPS) receivers, travel diaries and questionnaires. Separate multivariable logistic regression models were developed to examine factors associated with physical activity during the commute and mode of travel to work.

**Results:**

In comparison to car users (7.3 minutes±Standard Deviation 7.6), walkers (34.3±18.6) and public transport users (25.7±14.0) accrued substantially higher levels of daily moderate to vigorous physical activity during the commute. Combined accelerometer and GPS data showed that participants who walked at least ten minutes during their commute were more likely to have a shorter commute distance (p<0.001), occupy a sedentary job (p<0.01), and be classified as ‘underweight or normal weight’ (p<0.03). No car access (p<0.001), and absence of free work car parking (p<0.01) were independently related to walking to work and using public transport. Shorter commuting distances were also related to walking to work (p<0.001). Public transport users were more likely to be younger (p=0.04), have more positive environmental perceptions (p=0.01), and less likely to combine their commute with caring responsibilities (p=0.03).

**Conclusions:**

This study shows that walking to work and using public transport are important contributors to physical activity levels in a working population. Planning, transport and behavioural interventions to promote walking during the commute should take into account the wider determinants. Reducing availability of free work car parking is one possible strategy to discourage car use.

## Highlights

• Objective measures were used to characterise physical activity during the commute.
• Walkers and public transport users are more physically active than car users.
• Commute mode is influenced by individual characteristics and wider determinants.
• Reducing availability of free parking could help discourage car use.

## 1 Introduction

Global physical activity recommendations state that adults should accumulate at least 150 minutes of moderate physical activity per week in bouts of ten minutes or more to accrue health benefits (Haskell et al., 2007, World Health Organisation, 2010, Department of Health, 2011). However, there are concerns that due to increasing sedentary lifestyles many adults do not achieve this (Hallal et al., 2012). For example, in the United Kingdom (UK) 41% of adults aged 40 to 60 years old reported no occasions where they walk for ten minutes continuously at a brisk pace each month (Public Health England, 2017).

Evidence from systematic reviews suggests that adult populations who use active modes of transport (walking and cycling) for commuting have overall higher physical activity than car commuters, and also have decreased risk of cardiovascular disease and all-cause mortality (Hamer and Chida, 2008, Saunders et al., 2013, Kelly et al., 2014). Similarly, there is also evidence that people who use public transport, where a portion of the journey is by foot, accumulate more physical activity than car users (Wener and Evans, 2007, Lachapelle and Frank, 2009, Lachapelle and Noland, 2012, Rissel et al., 2012). The majority of primary studies have depended on self-report measures of both physical activity and mode of travel, which may not provide reliable estimates (Prince et al., 2008, Tully et al., 2014).

A number of studies have used objective methods (combining accelerometer or heart rate data and Global Positioning System (GPS) data) to investigate physical activity and mode of travel (Audrey et al., 2014, Costa et al., 2015, Miller et al., 2015, Audrey et al., 2015b). For example, in a population of 103 employees, those who walked to work accumulated more moderate to vigorous physical activity (MVPA) than car drivers on the days that they commuted (78.1 minutes per day, Standard Deviation (SD) 24.9 vs 49.8 minutes per day, SD 25.2), with no difference in weekend physical activity between the groups (Audrey et al., 2014, Audrey et al., 2015b).

Increasing the proportion of people who commute to work by walking or cycling has considerable potential to increase population-wide levels of physical activity, in addition to contributing to environmental benefits (British Medical Association, 2012). Despite being frequently combined as ‘active travel’, walking and cycling are discrete behaviours appealing to different population groups and requiring different strategies to increase their use as mode of travel (National Institute for Health and Care Excellence, 2012). However, walking may be perceived as an easier, safer and cheaper option, especially for those who are least active. Walking is a more familiar activity, does not require special equipment, and is less likely to involve direct competition for road space with motorised traffic (Morris and Hardman, 1997, Ogilvie et al., 2004). Because morbidity and mortality related to physical inactivity disproportionately affects socioeconomically deprived communities, encouraging and enabling walking as physical activity may help to address health inequalities.

In the UK, there are substantial opportunities to increase walking by replacing short journeys undertaken by car. For example, the 2016 National Travel Survey showed 24.5% of all car trips were shorter than two miles (3.2km), while 13% of trips of less than one mile (1.6km) were made by car (Department for Transport, 2017). Although levels of physical activity and personal travel modes may be considered a matter of individual choice, ecological models recognize the importance of a range of factors which constrain or support behaviour change. These have been conceptualized as operating at different levels: individual, interpersonal, organizational, community and public policy [14, 21].

Here, we examine baseline data from the Travel to Work multi-centre cluster randomised controlled trial (Audrey et al., 2015a). There are two aims for this cross-sectional study: (i) to examine, and compare by travel mode, the objectively measured physical activity of a working adult population, and, (ii) to identify associations between mode of travel to work and a range of individual, interpersonal, and organizational characteristics.

## 2 Materials and methods

### 2.1 Overview of the data collection methodology

We analysed baseline data obtained from 654 employees in 87 workplaces in urban areas of the south west of England and south Wales. Workplaces were recruited in two phases (May to July 2015 and March to May 2016). The methods of recruitment and sampling for the study have been described elsewhere in the study protocol paper (Audrey et al., 2015a). According to Office for National Statistics area classifications (Office for National Statistics 2011), the majority of recruited workplaces were located in ‘larger towns and cities’ (n=45, 51.7%), followed by ‘services, manufacturing and mining legacy’ (n=20, 23.0%) and ‘town living’ (n=13, 14.9%) areas. Workplaces were diverse in relation to their function and included public administration, professional and scientific organisations, retail, services and manufacturing. The workplaces also varied in size: 45 (51.7%) were small (fewer than 50 employees); 22 (25.3%) were medium (50–249 employees), and; 20 (23.0%) were large (250 or more employees).

Employees from participating workplaces were provided with information about the study and invited to participate. Those who provided written consent were asked to wear accelerometers (Actigraph GT3X+) for seven days during waking hours and a personal GPS receiver (QStarz BT1000XT), set to record positional data at ten second intervals, during their commute. They were also asked to complete travel diaries and questionnaires to collect individual and sociodemographic characteristics, psychological measures, factors relating to car use and perceptions of the commute. Participants who returned the equipment were provided with a £10 gift voucher to acknowledge their contribution to the study. Ethical approval for the study was obtained from the Faculty of Health Sciences Research Ethics Committee at the University of Bristol.

### 2.2 Objectively measured physical activity and main mode of travel during the commute

Raw accelerometer data were downloaded and summarised over ten second epochs for analysis using Actilife software (v6.11.8, ActiGraph LLC). Accelerometer data were then further processed using KineSoft (v3.3.80; KineSoft, Saskatchewan, Canada) data reduction software to generate outcome variables. Continuous periods of 60 minutes or more of zero values were considered ‘non-wear’ time and removed. To be included in the analysis of daily physical activity and sedentary behaviours, participants were required to provide at least three days of valid accelerometer data of at least 600 minutes duration. In relation to mode and physical activity during the commute, participants were required to provide at least one valid day of accGPS data on a working day. Days in which cycling was identified (either by accGPS data or travel diary data if not available) as the main mode of travel to work were excluded due to the inability of waist worn accelerometers to accurately record physical activity during cycling (Wetten et al., 2014).

The approach for data cleaning and assigning travel mode was adapted from a methodology developed for the PEACH study (Cooper et al. 2010). Accelerometer and GPS data were combined for every ten second epoch (accGPS) based upon the timestamp of the Actigraph data. We used predefined criteria during data cleaning to remove data points which were considered invalid or affected by signal loss (Cetateanu et al., 2016). Data points which were further than 500 m from any other GPS point or moving more than 100 km/ph were removed. For journeys where a loss of signal created a gap in the GPS trace of less than 25% and clearly the same journey to/from work, data was included. Where more than 25% was missing, data was excluded.

To ascertain trip origins and destinations, the participant's home and workplace were geocoded using the full postcode, and imported into a Geographical Information System (GIS) (ArcMap v10.2.2). The merged accGPS files were imported into ArcMap and participants outward and return journeys to work were visually identified and segmented from other accGPS data using the ‘identify’ tool. Journeys were identified as a continuous sequence of GPS locations between the participant's home and workplace, and therefore may include visits to other destinations (e.g. shopping) on the journey to/from work.

Mode of travel (walk, cycle, public transport or car) for the outward and return journeys over the measurement week was derived from visual analysis using the following variables: counts per ten seconds (sustained counts per epoch less than 17: bus, train, car; sustained counts per epoch greater than 325: walk and cycle (Freedson et al., 1998)); changes to sum of Signal to Noise Ratio (SNR) which describes the strength of the satellite signal through the ratio of the information content of a signal to its non-information content (threshold of drop to below 250 was employed to indicate movement from indoor to outdoor environment (Kerr et al., 2012)); maximum speed of the journey (walk: not greater than 10 km/hr; cycle: not greater than 40 km/hr; bus: 10 to 50 km/hr; train and car: speeds of greater than 50 km/hr (Stopher et al., 2008)), and; GIS location for each epoch.

Using the criteria described above, it was possible to identify changes in travel mode for different segments of the journey when participants used a mixed mode of travel (e.g. walk and train). Mixed mode of travel was not captured as a separate variable, instead the mode of transport of greatest distance was considered the mode for that journey. MVPA accrued from walking during the journey was captured in a separate variable. When an outward/return journey was missing, it was assumed to be the same mode of travel as the outward/return journey on the same day. Any remaining missing data were replaced with the corresponding travel diary mode where available. An overall mode of travel during data collection week for each participant was derived from the most frequently occurring mode of travel derived from each journey. Where different modes of travel occurred with equal frequency, we defined the overall mode of travel as the most active mode of travel.

Time spent being sedentary and in MVPA were defined using validated thresholds (sedentary less than 100 counts per minute (cpm); MVPA greater or equal to 1952 counts per minute) (Freedson et al., 1998). To examine the proportion of participants who met current physical activity guidelines (accruing greater or equal to 150 minutes per week of MVPA in bouts of ten minutes or more) (Department of Health, 2011), we calculated the total MVPA accumulated in bouts of ten minutes or more over the data collection week (by multiplying the mean daily bouted MVPA by seven). In line with another study (Panter et al., 2013), participants were classified as ‘active’ or ‘inactive’ during the commute if their mean daily MVPA accrued during the commute was ten minutes or greater, or less than ten minutes, respectively.

### 2.3 Variables: Individual characteristics and interpersonal responsibilities

The following variables were derived from questionnaire data: (i) gender; (ii) age group (‘below 35 years old’ or ‘35 years or greater’); (iii) annual household income (‘below £30,000’ or ‘£30,000 and above’ representing mean UK household income); (iv) level of education (‘degree or above’ or ‘below degree’); (v) occupational activity (‘sedentary’ or ‘non-sedentary’); (vi) no access to a car (absence of a current driving licence and/or household access to a car (‘yes’ or ‘no’)), and; (vii) combines commute with school run or caring responsibilities (‘yes’ or ‘no’). Self-reported height and weight were used to compute body mass index (BMI) and participants were assigned to either ‘normal or underweight’ (BMI less than 25 kg/m^2^) and ‘overweight or obese’ (BMI greater than or equal to 25 kg/m^2^) categories (World Health Organisation, 2000).

### 2.4 Variables: Workplace characteristics

In line with methods undertaken in our previous study (Audrey et al., 2014, Audrey et al., 2015b), commute distance was estimated using an online calculator (https://www.google.co.uk/maps) and the participant's home and work postcode. This was categorised as ‘two kilometres or below’, ‘between two and four kilometres’ and ‘four kilometres and above’. Participants were asked about the following policies and facilities at their workplace which might influence levels of walking during the commute: (i) free car parking; (ii) entitlement to purchase a car parking permit; (iii) secure storage for clothing; (iv) showers and changing rooms; (v) employer subsidised public transport schemes, and; (vi) travel plan or policy. Variables were categorised as ‘yes’ or ‘no’.

### 2.5 Variables: Perception of the commuting environment

To describe their perceptions of the commuting environment, participants stated how much they agreed with the following nine statements using a 5-point Likert scale: (i) ‘there are suitable pavements for walking’; (ii) ‘the pavements are well-maintained’; (iii) ‘there are not enough safe places to cross roads’; (iv) ‘walking is unsafe because of traffic’; (v) ‘it is unsafe because of the level of crime or antisocial behaviour’; (vi) ‘the routes for walking are generally well lit at night’; (vii) ‘the area is generally free from litter or graffiti’; (viii) ‘it is a pleasant environment for walking’, and; (ix) ‘there is a lot of air pollution’. These statements have been used in other studies (Panter et al., 2011, Panter et al., 2013, Adams et al., 2016) and have acceptable test-retest reliability (Ogilvie et al., 2008). In order for a high score to equate to agreement with the statement, negatively worded items were recoded. A mean substitution approach was used for the participants (n=7) who missed a single item on the scale. The distribution of scores was positively skewed. Therefore, a binary variable comprising ‘positive perception’ (less than mean score) and ‘negative perception’ (greater than or equal to mean score) was created.

### 2.6 Variables: Reasons for car use

To provide additional understanding of reasons for car use, participants whose main mode of travel was car were asked to indicate all reasons that applied to them from the following list: (i) quicker than alternatives; (ii) reliability; (iii) comfort; (iv) have to visit more than one place; (v) cheaper than alternatives; (vi) lack of alternative; (vii) personal safety; (viii) dropping off / collecting children; (ix) work unsociable hours; (x) car is essential to perform job; (xi) dropping off / collecting partner; (xii) carry bulky equipment; (xiii) health reasons; (xiv) giving someone else a lift, and; (xv) often on call. They were then asked to choose the single most important reason from the list.

### 2.7 Analysis

Initially, descriptive analyses comprising counts, percentages, medians and interquartile ranges, were performed. Differences in physical activity variables (overall and during the commute) were analysed by main mode of travel (car users, public transport users, and walkers) using Analysis of Variance (Anova) and chi-squared statistics. Data related to participants classified as cyclists are not presented because of the inability of waist worn accelerometers to accurately record physical activity during cycling.

### 2.8 Associations with objectively measured physical activity during the commute

To explore associations with levels of physical activity during the commute, logistic univariable analyses and likelihood ratio tests were performed. Based on our previous study (Audrey et al., 2014, Audrey et al., 2015b), the following explanatory variables for analysis were selected *a priori*: gender, age group, annual household income, education, weight status, occupational activity, and commute distance. A multivariable logistic regression model was developed using ‘inactive’ during commute as the reference group. In the order of the strength of association, variables were selected for inclusion and retained in the model if there was an associated improvement of fit (p<0.05). The final model adjusted for weight status, occupational activity, and commute distance.

### 2.9 Associations with mode of travel

The objective of the next stage of the analysis was to identify associations between different modes of travel to work and individual, interpersonal, and workplace variables. Analyses were restricted to participants who were classified as either ‘walkers’ (n=74), ‘public transport users’ (n=76) or ‘car users’ (n=422). Participants classified as cyclists (n=68) or whose mode of transport was unknown (n=14) were excluded. Initially, associations were examined using logistic univariable analyses and Likelihood Ratio Tests. Multicollinearity between variables was tested for through correlations. Using the same methodology as described previously, two separate multivariable logistic regression models were developed for ‘walkers’ and ‘public transport users’, both using ‘car users’ as the reference group. Individual-, interpersonal-, workplace- characteristics, and perception of commute variable were eligible for inclusion if they were associated with an improvement to fit of model (p<0.05). The final ‘walkers’ model adjusted for access to a car, commute distance, and availability of workplace car parking. The final ‘public transport users’ model adjusted for age group, access to a car, combines commute with caring responsibilities, availability of workplace car parking, and perception of commute environment.

Finally, a description of reasons for car use by car users was presented as counts and percentages.

Potential clustering by workplace was adjusted for using robust standard errors approach allowing for workplace-level random effects in the final model. For each model, results were presented as Odds Ratios (OR), adjusted Odds Ratios (aORs), and corresponding 95% Confidence Intervals (CIs) and p*-*values. Through sensitivity analyses, separate logistic models restricted to males only and females only were developed, with no major differences in effect sizes by variables observed (Supplementary Files). Interaction terms were not fitted due to small sample sizes. All analyses were performed with STATA statistical package, release 14 (STATA Corp, College Station, TX).

## 3 Results

Of the 654 participants who took part in the Travel to Work study at baseline, the majority were younger than 35 years old (431, 65.9%), and lived in a household with an income greater than £30,000 (455, 65.9%). There was a slight balance in favour of females (n=371, 56.7%) and having a degree (377, 57.7%) (Table 2).

**Table 1:** Objectively measured physical activity by main mode of travel to work.

	**All**	**Car**	**Walks**	**Public transport**	
	**N=540**	**N=357**	**N=62**	**N=62**	
	**n (%)**	**n (%)**	**n (%)**	**n (%)**	p-value*
					
Meets public health physical activity guidelines**	60 (11.1)	17 (4.8)	24 (38.7)	10 (16.1)	<0.001
	**Mean (SD)**	**Mean (SD)**	**Mean (SD)**	**Mean (SD)**	**p-value**^b^
					
Overall daily physical activity (counts per minute)	385.3 (193.1)	342.7 (120.1)	507.2 (151.2)	405.5 (150.7)	<0.001
Mean daily time spent in MVPA (minutes)	52.9 (28.7)	46.3 (20.6)	71.3 (21.3)	59.5 (26.6)	<0.001
Mean daily time spent in sedentary behaviours (minutes)	580.6 (72.6)	587.6 (69.5)	568.1 (62.2)	585.8 (65.2)	0.12
Mean daily wear time (minutes)	798.2 (75.7)	800.4 (75.0)	788.6 (70.9)	792.7 (72.2)	0.43
	**N=597**	**N=404**	**N=71**	**N=73**	
					
Mean daily commute time (minutes)	85.7 (54.2)	86.6 (51.0)	53.8 (29.2)	116.5 (56.2)	<0.001
Mean daily time spent in MVPA during commute (minutes)	13.0 (14.3)	7.3 (7.6)	34.3 (18.6)	25.7 (14.0)	<0.001

SD: Standard Deviation; MVPA: Moderate to Vigorous Physical Activity.

* Derived from chi-squared test;

b Derived from Analysis of Variance (Anova) statistics.

** Recommended physical activity for adults in 150 minutes accumulated throughout the week in bouts of at least 10 minutes.

**Table 2:** Univariable and multivariable model of predictors of incorporating some objectively measured physical activity during the commute.

	**All**	**Inactive**	**Active**	**OR (95% CIs)**	**p-value**	**aOR (95% CIs)**	**p-value**
	**N=654**	**N=349**	**N=248**				
	**n (%)**	**n (%)**	**n (%)**				
							
Gender: Male	283 (43.3)	160 (63.0)	94 (37.0)	–		NI	
Gender: Female	371 (56.7)	189 (55.1)	154 (44.9)	1.39 (1.00–1.93)	0.05		
Age group: Greater than 35 years old	431 (65.9)	241 (60.6)	157 (53.5)	–		NI	
Age group: Less than 35 years old	204 (31.2)	100 (39.5)	87 (46.5)	1.34 (0.94–1.90)	0.11		
Annual household income: Less than £30,000	121 (18.5)	65 (59.6)	44 (40.4)	–		NI	
Annual household income: Greater or equal to £30,000	455 (69.5)	243 (57.9)	177 (42.1)	1.08 (0.70–1.65)	0.74		
Education: Less than degree	247 (37.8)	138 (59.7)	93 (40.3)	–		NI	
Education: Degree or higher	377 (57.7)	195 (56.5)	150 (43.5)	1.14 (0.81–1.60)	0.44		
Weight status: Overweight or obese	296 (45.3)	179 (64.2)	100 (35.8)	–		–	
Weight status: Underweight or normal	293 (44.8)	143 (54.0)	122 (46.0)	1.53 (1.08–2.15)	0.02	1.48 (1.04–2.12)	0.03
Occupational activity: Non-sedentary	130 (19.9)	87 (69.1)	39 (31.0)	–		–	
Occupational activity: Sedentary	450 (68.8)	229 (55.5)	184 (44.6)	1.79 (1.17–2.74)	<0.01	1.96 (1.26–3.04)	<0.01
Commute distance: Greater than 4 km	455 (69.6)	276 (64.9)	149 (35.1)	–		–	
Commute distance: Between 2 km and 4 km	100 (15.3)	36 (40.9)	52 (59.1)	2.68 (1.67–4.28)	<0.001	2.73 (1.69–4.41)	<0.001
Commute distance: Less than 2 km	71 (10.9)	20 (40.6)	38 (59.4)	2.71 (1.58–4.63)	<0.001	2.74 (1.58–4.73)	<0.001

Inactive: Mean daily MVPA during commute<10 minutes; Active: Mean daily MVPA during commute≥10 minutes

aORs adjusted for weight status, occupational activity, and commute distance

NI: Not Included

Of 542 (82.4%) participants who provided three days of valid accelerometer data, a minority (n=60, 11.1%) met current UK public health physical activity guidelines (Department of Health, 2011). A substantially higher proportion of walkers (24, 38.7%) and public transport users (10, 16.1%) met public health physical activity guidelines than car users (17, 4.7%) (p<0.001). There were marked differences in time spent in MVPA by main mode of travel. Overall, both walkers (71.3 minutes, SD±21.3) and public transport users (59.5 minutes, SD±26.6) accumulated more MVPA throughout the day in comparison to car users (46.3 minutes, SD±20.6). Walkers (34.3 minutes, SD±18.6 minutes) and public transport users (25.7 minutes, SD±4.0) were also on average more active during the commute than car users (7.3 minutes, SD±7.6). There was no strong evidence for differences in time spent in sedentary behaviours (p=0.12) or accelerometer wear time (p=0.43) by main mode of travel (Table 2).

### 3.1 Associations with undertaking physical activity during the commute

At least one valid day of accGPS data was provided by 597 participants. After adjustment for weight status, occupational activity, and commute distance, there was strong evidence that participants were more physically active during their commute if they had sedentary jobs (aOR=1.96, 95% CI:1.26-3.04) or had a commute distance of less than two kilometres (aOR=2.73, 95% CI:1.69-4.41) or between two and four kilometres (aOR=2.74, 95% CI:1.58-4.73). There was weaker evidence that participants who belonged to the underweight or normal weight category (aOR=1.48, 95% CI:1.04-2.12) were more physically active during the commute (Table 1).

### 3.2 Individual characteristics, interpersonal responsibilities, workplace and environmental characteristics associated with mode of travel

After adjustment for access to a car, commute distance to workplace, and free work car parking, there was strong evidence that not having access to a car (aOR=20.4, 95% CI:6.01-69.8) was positively associated with walking to work. Workplace characteristics ‘commute distance of less than two kilometres’ (aOR=63.6, 95% CI:21.5-187.9), ‘commute distance between two kilometres and four kilometres’ (aOR=15.0, 95% CI:5.55-40.6), lack of free parking (aOR=3.19, 95% CI:1.38-7.39) were also positively associated with walking (Table 3).

**Table 3:** Univariable and multivariable model of predictors associated with walking and public transport as main mode of travel to work.

	**All**	**Car users**	**Walkers**					**Public transport users**				
	**N=654**	**N=422**	**N=74**					**N=76**				
***Individual & interpersonal***	**n (%)**	**n (%)**	**n (%)**	**OR (95% CI)**	**p-value**	**aOR (95% CI)**	**p-value**	**n (%)**	**OR (95% CI)**	**p-value**	**aOR (95% CI)**	**p-value**
Gender: Female	371 (56.7)	241 (57.1)	44 (59.5)	1.10 (0.67–1.82)	0.71	NI		50 (65.8)	1.44 (0.87–2.41)	0.16	NI	
												
Age group:	204 (31.2)	115 (27.3)	28 (37.8)	1.69 (1.00–2.85)	0.05	NI		38 (50.0)	2.67 (1.62–4.41)	<0.001	2.05 (1.05–4.02)	0.04
Less than 35 years old												
												
Annual household income:	121 (18.5)	80 (19.0)	17 (23.0)	1.34 (0.73–2.46)	0.34	NI		12 (15.8)	0.84 (0.43–1.65)	0.61	NI	
Less than £30,000												
												
Education:	247 (37.8)	169 (40.1)	32 (43.2)	1.15 (0.69–1.90)	0.60	NI		29 (38.2)	0.88 (0.53–1.46)	0.62	NI	
Less than degree												
												
Weight status:	293 (44.8)	178 (42.2)	44 (59.5)	2.31 (1.34–3.97)	<0.01	NI		28 (36.8)	0.99 (0.58–1.70)	0.99	NI	
Underweight or normal												
												
Occupational activity:	130 (19.9)	288 (68.3)	50 (67.6)	1.39 (0.71–2.72)	0.34	NI		61 (80.3)	2.54 (1.17–5.50)	0.02	NI	
Sedentary												
Access to car: No	62 (9.5)	9 (2.1)	21 (28.4)	20.4 (8.78–47.2)	<0.001	20.5 (6.01–69.8)	<0.001	27 (35.5)	26.8 (11.8–60.7)	<0.001	29.2 (10.4–81.6)	<0.001
Combines commute with caring responsibilities: No	485 (74.2)	308 (73.0)	59 (79.7)	2.52 (1.05–6.05)	0.04	NI		64 (84.2)	5.47 (1.67–17.9)	<0.01	4.88 (1.17–20.3)	0.03
												
***Workplace***												
Commute distance: Greater than 4 km	–	–	–	–	–	–	–	–	–	–	–	
												
Commute distance:	100 (15.3)	48 (11.4)	21 (28.4)	11.3 (5.30–24.0)	<0.001	15.0 (5.55–40.6)	<0.001	8 (10.5)	0.87 (0.39–1.93)	0.74	NI	
Between 2 and 4km												
												
Commute distance:	71 (10.9)	26 (6.2)	35 (47.3)	34.7 (16.4–73.5)	<0.001	63.6 (21.5–187.9)	<0.001	1 (1.3)	0.21 (0.03–1.60)	0.13	NI	
2km and less												
Absence of free work car parking	253 (38.7)	147 (34.8)	38 (51.4)	3.02 (1.69–5.40)	<0.001	3.19 (1.38–7.39)	<0.01	38 (50.0)	2.88 (1.63–5.10)	<0.001	3.81 (1.75–8.27)	<0.01
No entitlement to purchase a parking permit	444 (67.9)	299 (70.9)	49 (66.2)	1.62 (0.70–3.72)	0.26	NI		46 (60.5)	1.33 (0.60–2.94)	0.49	NI	
Secure storage for personal belongings	218 (52.0)	217 (58.3)	39 (65.0)	1.33 (0.75–2.34)	0.33	NI		37 (59.7)	1.06 (0.61–1.82)	0.84	NI	
Absence of showers and changing rooms	195 (29.8)	131 (31.0)	25 (33.8)	1.22 (0.71–2.11)	0.48	NI		24 (39.3)	1.20 (0.69–2.10)	0.51	NI	
No employer subsidised public transport schemes	406 (11.2)	268 (62.1)	42 (56.8)	2.15 (0.74–6.26)	0.16	NI		48 (63.2)	1.23 (0.55–2.75)	0.61	NI	
Absence of Travel plan or policy	233 (35.6)	151 (35.8)	25 (33.8)	1.77 (0.79–3.94)	0.17	NI		35 (46.1)	1.71 (0.86–3.40)	0.13	NI	
												
**Environmental**												
Positive perception	290 (44.3)	161 (38.2)	46 (62.2)	3.44 (1.95–6.10)	<0.001	NI		19 (25.0)	2.35 (1.39–3.98)	<0.01	2.71 (1.24–5.92)	0.01

Comparator group: Car users; NI: Not included in model

aWalkers aORs adjusted for workplace, access to car, distance to workplace, and absence of free work car parking;

Public transport aORs adjusted for workplace, age group, access to car, combines commute with caring responsibilities, and absence of free work car parking

After adjustment for age group, access to car, combining commute with caring responsibilities, free work car parking and perception of commute environment, there was strong evidence that being aged less than 35 years old (aOR=2.05, 95% CI:1.05-4.02), not having access to a car (aOR=29.2, 95% CI:10.4-81.6), not combining the commute with school run or caring responsibilities (aOR=4.88, 95% CI:1.17-20.3) were positively associated with using public transport. The workplace characteristic ‘absence of free parking’ (aOR=3.81, 95% CI:1.75-8.27) and more positive perceptions of the commute environment (aOR=2.71, 95% CI:1.24-5.92) were also positively associated with using public transport (Table 3).

Sensitivity analyses indicated no marked differences by gender across all the models.

### 3.3 Reasons for car use

Reasons frequently provided for car use included being quicker than alternatives (n=329, 78.0%), reliability (275, 65.2%), comfort (275, 65.2%), having to visit more than one place (179 (42.4%), cheaper than alternatives (179, 42.2%), and lack of alternatives (174, 41.2%). This order changed slightly when participants were asked to choose a single most important reason. Quicker than alternatives (n=100, 28.7%), lack of alternatives (51, 14.7%), and reliability (39, 11.2%) continued as main reasons, but dropping off and collecting children (34, 9.8%) and a car being essential for the job (29, 8.3%) appeared to take priority over comfort, cost or having to visit more than one place.

## 4 Discussion

We confirm low levels of objectively measured physical activity in a UK adult working population. Strong associations of physical activity with both walking and public transport as the main mode of travel to work were also observed. Similar to other studies reporting objective measures of physical activity (Hagstromer et al., 2007, Craig et al., 2009, Mutikainen et al., 2014), a low proportion of participants in our study (~11%) met current physical activity recommendations (Department of Health, 2011). The current study (undertaken with a larger sample of employees recruited from geographically distinct areas) builds on the findings of previous work which found that walking to work was an important contributor to overall physical activity levels (Audrey et al., 2014, Audrey et al., 2015b).

We were also able to detect lower levels of physical activity during the commute in participants who held non-sedentary jobs and those who lived further away from their workplace. We speculate that the association between non-sedentary work and less physical activity may be because those who believe they have been physically active during the working day, even though they are not achieving sufficient levels of physical activity to achieve health benefits, may be less inclined to undertake additional physical activity during the commute. This requires further investigation but we would suggest, in addition to population-wide interventions to increase active travel, there is a need for specific interventions targeting population groups less likely to engage in physical activity (Winters et al., 2017).

Given the potential benefits of incorporating walking within the commute, we examined factors at different levels of the socioecological model which are likely to support this behaviour. In line with the findings of other UK-based studies (Panter et al., 2011, Dalton et al., 2013), personal factors related to walking to work or using public transport included not having access to a car or a driving licence. Previous qualitative research has suggested that childcare commitments may restrict choice of travel mode for the commute (Faulkner et al., 2010, Jain et al., 2011). Within our sample of employees, we found that public transport users were also less likely to incorporate interpersonal commitments such as taking children to school or other caring responsibilities within their commute, however there was no evidence of this association for walkers.

We found walking to work to be positively associated with a shorter commuting distance and a lack of free car parking at work. Public transport use was also associated with lack of free parking. Other workplace travel plans or policies, incentives (subsidised public transport, entitlement to purchase parking permits) or workplace facilities (secure storage, showers, changing rooms) did not appear to be associated with increased walking or public transport use. Such initiatives are often included in sustainable travel plans, and may be regarded by employers and employees as easier to implement than more radical policies such as removing car parks or encouraging people to live closer to their workplaces. Future studies evaluating the implementation of different types of workplace policies could be undertaken to establish whether such approaches can be effective in changing travel behaviours.

In terms of a shorter commuting distance, our results add weight to the argument that more active modes of travel, and reductions in private car use, can be encouraged by local and national government policies which provide and maintain employment opportunities close to where people live (Department of Communities and Local Government, 2012). As regards free on-site parking, the removal of this ‘perk’ is unlikely to be popular with employees who value driving to work. Previous qualitative research has suggested that, where removing parking might be perceived as punitive, employers would prefer this to be imposed from outside of the workplace (Audrey and Procter, 2015). This might, for example, be a directive from a more distant ‘head office’ or because of policies imposed by local or national government.

In other UK-based studies short distance to workplace (Panter et al., 2011, Dalton et al., 2013, Panter et al., 2013) and a lack of onsite car parking at work (Panter et al., 2011, Dalton et al., 2013, Procter et al., 2014, Audrey and Procter, 2015) have been shown to act as facilitators of walking travel modes. In the current study, the majority of participants had a commute distance greater than two kilometres (n=555, 84.8%). Therefore, switching to walking as the main mode of travel to work may not be feasible for many of our study population. However, a mixed mode commute, combining walking with public transport, may be possible. A case study of 20 UK workplaces showed that limiting parking, either by introducing parking charges or reducing spaces available, and providing payments for public transport users, was a critical factor contributing to decreased car use (Cairns et al., 2010). The implementation of workplace policies to limit or charge for workplace car parking, whilst offering subsidies for public transport, may make modes of travel other than a car more appealing. The effects of how such workplace policies translate into changes of walking, cycling and physical activity levels require further evaluation to provide evidence for practitioners and policy-makers.

We found the main reason given by car users for their travel mode during the commute was that it was quicker than alternatives. Other important reasons included reliability and lack of alternatives, which suggests that in the study locations the public transport infrastructure may not be perceived as efficient enough for daily use. This implies that behavioural interventions and workplace strategies to discourage car use would also need to be supported by transport and planning policies to make public transport a more acceptable choice for those who need to travel longer distances for work (Table 4).

**Table 4:** Reasons for mode of travel by car users.

**Ordered by most important reason**		**Ordered by all reasons**		
	**N=348**		**N=383**	
	**n (%)**		**n (%)**	
Quicker than alternatives	100 (28.7)	Quicker than alternatives	329 (78.0)	
Lack of alternative	51 (14.7)	Reliability	275 (65.2)	
Reliability	39 (11.2)	Comfort	275 (65.2)	
Dropping off/collecting children	34 (9.8)	Have to visit more than one place	179 (42.4)	
Car is essential to perform job	29 (8.3)	Cheaper than alternatives	178 (42.2)	
Have to visit more than one place	16 (4.6)	Lack of alternative	174 (41.2)	
Cheaper than alternatives	15 (4.3)	Personal safety	115 (27.3)	
Dropping off/collecting partner	14 (4.0)	Dropping off/collecting children	103 (24.4)	
Comfort	13 (3.7)	Work unsociable hours	99 (23.5)	
Work unsociable hours	10 (2.9)	Car is essential to perform job	91 (21.6)	
Carry bulky equipment and/or cash	9 (2.6)	Dropping off/collecting partner	38 (9.0)	
Health reasons	6 (1.7)	Carry bulky equipment and/or cash	80 (19.0)	
Personal safety	5 (1.4)	Health reasons	23 (5.5)	
Giving someone else a lift	4 (1.2)	Giving someone else a lift	55 (13.0)	
Often on call	3 (0.9)	Often on call	28 (6.6)	

Univariable analyses suggested both walkers and public transport users had more positive perceptions of their commute environment than car users. After adjustment for other variables this positive perception of the commute environment remained evident for public transport users but not for walkers. This showed that other factors, such as not having a driving license and commute distance, are much more important and overriding in relation to walking to work for our study population. Guell *et al.* suggest that some participants will walk despite adverse environmental conditions, having overcome the issue through experience or weighing up the perceived benefits and costs (Guell et al., 2013).

As we did not objectively quantify characteristics of the environment through GIS-based measures, we cannot eliminate whether there were physical differences in relation to the commute environment. Cross-sectional data from other studies indicates positive associations between walking and characteristics of the built environment including street connectivity and pedestrian lanes (Saelens and Handy, 2008, McCormack and Shiell, 2011). This does suggest that, amongst other measures, car drivers may need more positive perceptions of their commuting route if they are to be persuaded to change their travel mode. In the UK, nationwide construction of walking and cycling routes has been shown to be associated with increased physical activity levels and walking and cycling as modes of travel (Goodman et al., 2014, Panter and Ogilvie, 2017). However, distance to the infrastructure was identified as a mediating factor, suggesting that fragmented improvements to infrastructure may not be sufficient to bring about behaviour changes (Song et al., 2017).

### 4.1 Strengths and limitations

There are several noteworthy strengths of this study. To our knowledge, this is the largest study that has combined robust, objectively measured data derived from accelerometer and GPS devices to identify characteristics associated with physical activity and walking as the main mode of travel to work. Data were collected over a relatively short period of time (May to July 2015 and March to May 2016) to minimise the effect of seasonality on physical activity and travel behaviours. In this study, 65% and 11% of the study participants were categorised as car users and walkers respectively. Similarly, findings from the National Travel Survey (England 2016) showed that 64% and 11% of commute journeys were made by car and walking respectively (Department for Transport, 2017). This suggests that the findings we report are similar to national patterns of commuting behaviour and could be more widely generalisable.

There are some limitations to the study. As analyses were undertaken on cross-sectional data, we are unable to establish causal relationships between the variables of interest. Participants were classified according to their overall mode of travel derived from the most frequently occurring mode in each journey during the data collection week. Participants who used mixed modes of travel were not systematically identified as this was not an outcome measure for the main study trial (Audrey et al., 2015a). Therefore, error may have been introduced to the models as a result of participants being classified by a single travel mode of travel. We used perceptions of the commute environment, rather than objectively quantified characteristics of the environment through a GIS. Participants of the study were relatively young, predominantly well-educated and employed in sedentary occupations. Therefore, the findings of this study may not be applicable to a population with different characteristics. Other factors not analysed in this study may also affect travel choices, such as access to public transport, psychological measures, and perceptions of the residential or workplace neighbourhood.

## 5 Conclusions

We have shown that walking to work, either the whole route or combined with public transport, is an important contributor to objectively measured physical activity levels in a large sample of adult employees recruited from diverse workplaces and settings in the UK. Interventions to increase walking to work should take into account individual and wider determinants of commuting behaviour. At an individual level these include access to a car and driving licence. At organisational and policy levels, consideration needs to focus on commuting distances, availability of car parking, and the availability of alternative modes of transport. Our study has contributed to, and enhanced, what is already known. We believe the picture is sufficiently clear to assert that supporting walking during the daily commute (either as the main mode or as part of a mixed-mode journey) should be a priority for both transport and public health disciplines.
