# Supplementary material for: Associations of mode of travel to work with physical activity, and individual, interpersonal, organisational, and environmental characteristics
Source: J Transp Health. 2018 Jun;9:45–55. doi: 10.1016/j.jth.2018.01.009 (PMC6011385; doi:10.1016/j.jth.2018.01.009)
Supplement: Supplementary file 1 — Supplementary material [file mmc1.docx]

| *Supplementary Table 1. Univariable and multivariable model of predictors of incorporating some objectively measured physical activity during the commute Males only* | | | | | | | | | |
| --- | --- | --- | --- | --- | --- | --- | --- | --- | --- |
|  | **Males** | **Inactive** | **Active** | **OR (95% CIs)** | **p-value** | **aOR (95% CIs)** | **p-value** |  |  |
|  | **N=283** | **N=349** | **N=248** |  |  |  |  |  |  |
|  | **n (%)** | **n (%)** | **n (%)** |  |  |  |  |  |  |
| Age group: Greater than 35 years old | 179 (63.3) | 108 (65.9) | 56 (34.2) | - |  | NI |  |  |  |
| Age group: Less than 35 years old | 91 (32.2) | 47 (57.3) | 35 (42.7) | 1.44 (0.83-2.47) | 0.19 |  |  |  |  |
| Annual household income: Less than £30,000 | 44 (15.6) | 25 (62.5) | 15 (37.5) | - |  | NI |  |  |  |
| Annual household income: Greater or equal to £30,000 | 203 (71.7) | 116 (63.4) | 67 (36.6) | 0.96 (0.47-1.95) | 0.92 |  |  |  |  |
| Education: Less than degree | 98 (34.6) | 61 (67.8) | 29 (32.2) | - |  | NI |  |  |  |
| Education: Degree or higher | 164 (58.0) | 87 (58.8) | 61 (41.2) | 1.47 (0.85-2.56) | 0.17 |  |  |  |  |
| Weight status: Overweight or obese | 144 (50.9) | 90 (67.2) | 44 (32.8) | - |  | - |  |  |  |
| Weight status: Underweight or normal | 109 (38.5) | 57 (59.4) | 39 (40.6) | 1.40 (0.81-2.41) | 0.23 | NI |  |  |  |
| Occupational activity: Non-sedentary | 55 (19.4) | 39 (72.2) | 15 (27.8) | - |  | - |  |  |  |
| Occupational activity: Sedentary | 191 (67.5) | 103 (60.6) | 67 (39.4) | 1.69 (0.87-3.31) | 0.12 | NI |  |  |  |
| Commute distance: Greater than 4km | 197 (69.6) | 124 (68.5) | 57 (31.5) | - |  | - |  |  |  |
| Commute distance: Between 2km and 4km | 36 (12.7) | 16 (51.6) | 15 (48.4) | 2.04 (0.94-4.41) | 0.07 | 2.04 (0.94-4.41) | 0.07 |  |  |
| Commute distance: Less than 2km | 33 (11.7) | 14 (45.2) | 17 (54.8) | 2.64 (1.22-5.73) | 0.01 | 2.64 (1.22-5.73) | 0.01 |  |  |
| Inactive: Mean daily MVPA during commute<10 minutes; Active: Mean daily MVPA during commute≥10minutes  aORs adjusted for commute distance  NI: Not Included | | | | | | | |  |  |

| *Supplementary Table 2. Univariable and multivariable model of predictors of incorporating some objectively measured physical activity during the commute Females only* | | | | | | | |  |
| --- | --- | --- | --- | --- | --- | --- | --- | --- |
|  | **Females** | **Inactive** | **Active** | **OR (95% CIs)** | **p-value** | **aOR (95% CIs)** | **p-value** | |
|  | **N=371** | **N=349** | **N=248** |  |  |  |  | |
|  | **n (%)** | **n (%)** | **n (%)** |  |  |  |  | |
| Age group: Greater than 35 years old | 252 (67.9) | 133 (56.8) | 101 (43.2) | - |  | NI |  | |
| Age group: Less than 35 years old | 113 (30.5) | 100 (39.5) | 87 (46.5) | 1.29 (0.81-2.05) | 0.28 |  |  | |
| Annual household income: Less than £30,000 | 77 (20.8) | 40 (58.0) | 29 (42.0) | - |  | NI |  | |
| Annual household income: Greater or equal to £30,000 | 252 (67.9) | 127 (53.6) | 110 (46.4) | 1.08 (0.70-1.65) | 0.74 |  |  | |
| Education: Less than degree | 149 (40.2) | 77 (54.6) | 64 (45.4) | - |  | NI |  | |
| Education: Degree or higher | 213 (57.4) | 108 (54.8) | 89 (45.2) | 0.99 (0.64-1.43) | 0.97 |  |  | |
| Weight status: Overweight or obese | 152 (41.0) | 89 (61.4) | 56 (38.6) | - |  | NI |  | |
| Weight status: Underweight or normal | 184 (49.6) | 86 (50.9) | 83 (49.1) | 1.53 (0.98-2.41) | 0.06 |  |  | |
| Occupational activity: Non-sedentary | 75 (20.2) | 48 (66.7) | 24 (33.3) | - |  | - |  | |
| Occupational activity: Sedentary | 259 (69.8) | 126 (51.9) | 117 (48.2) | 1.86 (1.07-3.22) | 0.03 | 1.98 (1.11-3.51) | 0.02 | |
| Commute distance: Greater than 4km | 258 (69.5) | 152 (62.3) | 92 (37.7) | - |  | - |  | |
| Commute distance: Between 2km and 4km | 64 (17.3) | 20 (35.1) | 37 (64.9) | 2.68 (1.67-4.28) | <0.001 | 3.24 (1.76-5.99) | <0.001 | |
| Commute distance: Less than 2km | 38 (10.2) | 12 (36.4) | 21 (63.6) | 2.71 (1.58-4.63) | <0.001 | 2.81 (1.31-6.02) | <0.01 | |
| Inactive: Mean daily MVPA during commute<10 minutes; Active: Mean daily MVPA during commute≥10minutes  aORs adjusted for occupational activity, and commute distance  NI: Not Included | | | | | | | | |

| *Supplementary Table 3. Univariable and multivariable model of predictors associated with walking and public transport as main mode of travel to work Males only* | | | | | | | | | | | | | | | | | | | | | |
| --- | --- | --- | --- | --- | --- | --- | --- | --- | --- | --- | --- | --- | --- | --- | --- | --- | --- | --- | --- | --- | --- |
|  | **Males** | **Car users** | **Walkers** | | | | | |  | **Public transport users** | | | | | | | | | | | |
|  | **N=207** | **N=181** | **N=30** |  |  | |  | |  | **N=26** |  | | |  | |  | |  | | |  |
| ***Individual & interpersonal*** | **n (%)** | **n (%)** | **n (%)** | **OR (95% CI)** | **p-value** | | **aOR (95% CI)** | | **p-value** | **n (%)** | **OR (95% CI)** | | | **p-value** | | **aOR (95% CI)** | | | **p-value** | |  |
| Gender: N/A |  |  |  |  |  | |  | |  |  |  | | |  | |  | | |  | |  |
| Age group:  Less than 35 years old | 91 (32.2) | 48 (26.5) | 14 (46.7) | 2.87 (1.26-6.55) | 0.01 | | NI | |  | 12 (46.2) | 2.29 (0.99-5.29) | | | 0.05 | | NI | | |  | |  |
| Annual household income:  Less than £30,000 | 44 (15.6) | 30 (16.6) | 7 (23.3) | 1.78 (0.68-4.69) | 0.24 | | NI | |  | 2 (7.7) | 2.54 (0.57-11.4) | | | 0.22 | | NI | | |  | |  |
| Education:  Less than degree | 98 (34.6) | 70 (38.7) | 11 (36.7) | 0.96 (0.42-2.20) | 0.93 | | NI | |  | 7 (26.9) | 1.94 (0.77-4.86) | | | 0.16 | | NI | | |  | |  |
| Weight status:  Underweight or normal | 109 (38.5) | 63 (34.8) | 14 (46.7) | 2.08 (0.89-4.87) | 0.09 | | NI | |  | 9 (34.6) | 1.05 (0.43-2.57) | | | 0.91 | | NI | | |  | |  |
| Occupational activity:  Sedentary | 144 (69.6) | 120 (66.3) | 17 (56.7) | 1.49 (0.47-4.67) | 0.50 | | NI | |  | 24 (92.3) | 8.40 (1.10-64.0) | | | 0.04 | | NI | | |  | |  |
| Limited access to car: No | 21 (7.4) | 4 (2.2) | 7 (23.3) | 17.4 (4.59-65.9) | <0.001 | | 22.0 (2.91-165.0) | | <0.01 | 7 (26.9) | 15.5 (4.12-58.0) | | | <0.001 | | 17.3 (5.57-53.9) | | | <0.001 | |  |
| Combines commute with caring responsibilities: Yes | 36 (12.7) | 24 (13.3) | 2 (6.7) | 1.91 (0.42-8.67) | 0.40 | | NI | |  | 24 (92.3) | 3.40 (0.44-26.6) | | | 0.24 | | NI | |  | | |  |
| ***Workplace*** |  |  |  |  |  | |  | |  |  |  | | |  | |  | |  | | |  |
| Commute distance: Greater than 4km | 197 (69.6) | - | - | - | - | | - | | - | - | - | | | - | | - | |  | | |  |
| Commute distance:  Between 2 and 4 kilometres | 36 (12.7) | 15 (8.3) | 5 (16.7) | 12.1 (2.93-49.9) | 0.01 | | 9.67 (1.68-55.7) | | 0.01 | 3 (11.5) | 1.26 (0.34-4.70) | | | 0.73 | | NI | |  | | |  |
| Commute distance:  2 kilometres and less | 33 (11.7) | 13 (7.2) | 17 (56.7) | 47.4 (13.9-161.9) | <0.001 | | 60.7 (14.2-260.1) | | <0.001 | 0 (0.0) | NI | | | NI | | NI | |  | | |  |
| Absence of free work car parking | 85 (30.0) | 39 (21.6) | 15 (50.0) | 6.37 (2.42-16.8) | <0.001 | | 4.41 (1.23-15.8) | | 0.02 | 15 (57.7) | 5.58 (2.20-14.2) | | | <0.001 | | 1.80 (0.83-3.94) | | 0.14 | | |  |
| No entitlement to purchase a parking permit | 199 (70.3) | 130 (71.8) | 19 (63.3) | 1.39 (0.30-6.44) | 0.68 | | NI | |  | 18 (69.2) | 0.88 (0.24-3.26) | | | 0.85 | | NI | |  | | |  |
| Secure storage for personal belongings | 141 (49.8) | 88 (48.6) | 13 (43.3) | 1.39 (0.53-3.68) | 0.50 | | NI | |  | 14 (53.9) | 1.05 (0.44-2.51) | | | 0.91 | | NI | |  | | |  |
| Absence of showers and changing rooms | 64 (22.6) | 42 (23.2) | 8 (26.7) | 1.55 (0.61-3.96) | 0.36 | | NI | |  | 7 (26.9) | 1.19 (0.46-3.09) | | | 0.73 | | NI | |  | | |  |
| No employer subsidised public transport schemes | 172 (60.8) | 110 (60.7) | 16 (53.3) | 3.49 (0.44-27.6) | 0.24 | | NI | |  | 18 (69.2) | 1.31 (0.36-4.80) | | | 0.69 | | NI | |  | | |  |
| Absence of Travel plan or policy | 95 (33.6) | 61 (33.7) | 9 (30.0) | 1.40 (0.40-4.87) | 0.60 | | NI | |  | 11 (42.3) | 1.14 (0.39-3.34) | | | 0.81 | | NI | |  | | |  |
| **Environmental** |  |  |  |  |  | |  | |  |  |  | | |  | |  | |  | | |  |
| Positive perception | 125 (44.2) | 62 (34.3) | 19 (63.3) | 7.58 (2.46-23.3) | <0.001 | | NI | |  | 17 (65.4) | 3.88 (1.52-9.88) | | | <0.01 | | NI | |  | | |  |
| Comparator group: Car users; NI: Not included in model | | | | | |  | |  | | | |  |  | |  | |  | | |  |  |
| Walkers aORs adjusted for limited access to car, distance to workplace, and absence of free work car parking;  Public transport aORs adjusted for limited access to car, and absence of free work car parking | | | | | | | | | | | | | | | | | | | |  |  |

| *Supplementary Table 4. Univariable and multivariable model of predictors associated with walking and public transport as main mode of travel to work Females only* | | | | | | | | | | | | | | | | | | | | | |
| --- | --- | --- | --- | --- | --- | --- | --- | --- | --- | --- | --- | --- | --- | --- | --- | --- | --- | --- | --- | --- | --- |
|  | **Females** | **Car users** | **Walkers** | | | | | |  | **Public transport users** | | | | | | | | | | | |
|  | **N=371** | **N=241** | **N=44** |  |  | |  | |  | **N=50** |  | | |  | |  | |  | | |  |
| ***Individual & interpersonal*** | **n (%)** | **n (%)** | **n (%)** | **OR (95% CI)** | **p-value** | | **aOR (95% CI)** | | **p-value** | **n (%)** | **OR (95% CI)** | | | **p-value** | | **aOR (95% CI)** | | | **p-value** | |  |
| Gender: N/A |  |  |  |  |  | |  | |  |  |  | | |  | |  | | |  | |  |
| Age group:  Less than 35 years old | 113 (30.5) | 67 (27.8) | 14 (31.8) | 1.19 (0.59-2.39) | 0.62 | | NI | |  | 26 (52.0) | 2.89 (1.54-5.41) | | | <0.01 | | 2.75 (1.24-6.10) | | | 0.01 | |  |
| Annual household income:  Less than £30,000 | 77 (20.8) | 50 (20.8) | 10 (22.7) | 1.11 (0.51-2.43) | 0.79 | | NI | |  | 10 (20.0) | 1.08 (0.49-2.35) | | | 0.85 | | NI | | |  | |  |
| Education:  Less than degree | 149 (40.2) | 99 (41.1) | 21 (47.7) | 1.27 (0.67-2.43) | 0.46 | | NI | |  | 22 (44.0) | 1.14 (0.61-2.11) | | | 0.69 | | NI | | |  | |  |
| Weight status:  Underweight or normal | 184 (49.6) | 115 (47.7) | 30 (68.2) | 2.43 (1.19-5.00) | 0.02 | | NI | |  | 19 (38.0) | 0.93 (0.47-1.83) | | | 0.82 | | NI | | |  | |  |
| Occupational activity:  Sedentary | 259 (69.8) | 168 (69.7) | 33 (75.0) | 1.33 (0.58-3.04) | 0.51 | | NI | |  | 37 (74.0) | 1.70 (0.72-4.03) | | | 0.23 | | NI | | |  | |  |
| Limited access to car | 41 (11.1) | 5 (2.1) | 14 (31.8) | 22.5 (7.53-67.2) | <0.001 | | 52.6 (10.2-270.7) | | <0.001 | 20 (40.0) | 36.0 (12.4-104.3) | | | <0.001 | | 33.7 (9.90-114.5) | | | <0.001 | |  |
| Combines commute with caring responsibilities: No | 250 (67.4) | 150 (62.2) | 38 (86.4) | 4.88 (1.68-14.2) | <0.01 | | 7.62 (1.77-32.7) | | <0.01 | 38 (76.0) | 3.90 (1.48-10.3) | | | <0.01 | | 5.31 (1.52-18.6) | | <0.01 | | |  |
| ***Workplace*** |  |  |  |  |  | |  | |  |  |  | | |  | |  | |  | | |  |
| Commute distance: Greater than 4km | 258 (69.5) | - | - | - | - | | - | | - | - | - | | | - | | - | |  | | |  |
| Commute distance:  Between 2 and 4 kilometres | 64 (17.3) | 33 (13.7) | 16 (36.4) | 10.2 (4.18-25.1) | <0.001 | | 11.1 (3.92-31.5) | | <0.01 | 5 (10.0) | 0.70 (0.26-1.91) | | | 0.49 | | NI | |  | | |  |
| Commute distance:  2 kilometres and less | 38 (10.2) | 13 (5.4) | 18 (40.9) | 29.2 (11.0-77.7) | <0.001 | | 31.3 (9.49-103.1) | | <0.001 | 1 (2.0) | 0.36 (0.05-2.80) | | | 0.33 | | NI | |  | | |  |
| Absence of free work car parking | 168 (45.3) | 108 (44.8) | 23 (52.3) | 1.93 (0.93-4.0) | 0.08 | | 3.19 (1.38-7.39) | | <0.01 | 23 (46.0) | 1.93 (0.93-4.01) | | | 0.08 | | 5.81 (1.74-19.4) | | 0.01 | | |  |
| No entitlement to purchase a parking permit | 245 (66.0) | 169 (70.1) | 30 (68.2) | 1.78 (0.65-4.82) | 0.26 | | NI | |  | 28 (56.0) | 1.66 (0.61-4.52) | | | 0.32 | | NI | |  | | |  |
| Secure storage for personal belongings | 199 (53.6) | 129 (53.5) | 26 (59.1) | 1.28 (0.63-2.59) | 0.49 | | NI | |  | 23 (46.0) | 1.06 (0.52-2.14) | | | 0.88 | | NI | |  | | |  |
| Absence of showers and changing rooms | 131 (35.3) | 89 (36.9) | 17 (38.6) | 1.03 (0.52-2.02) | 0.94 | | NI | |  | 17 (34.0) | 1.17 (0.59-2.35) | | | 0.65 | | NI | |  | | |  |
| No employer subsidised public transport schemes | 234 (63.1) | 158 (65.6) | 26 (59.1) | 1.70 (0.48-5.97) | 0.41 | | NI | |  | 30 (60.0) | 1.18 (0.42-3.27) | | | 0.75 | | NI | |  | | |  |
| Absence of Travel plan or policy | 138 (37.2) | 90 (37.3) | 16 (36.4) | 2.06 (0.72-5.93) | 0.18 | | NI | |  | 24 (48.0) | 2.21 (0.89-5.46) | | | 0.09 | | NI | |  | | |  |
| **Environmental** |  |  |  |  |  | |  | |  |  |  | | |  | |  | |  | | |  |
| Positive perception | 165 (44.5) | 99 (41.1) | 27 (61.4) | 2.36 (1.19-4.68) | 0.01 | | NI | |  | 26 (52.0) | 1.80 (0.94-3.43) | | | 0.08 | | NI | |  | | |  |
| Comparator group: Car users; NI: Not included in model | | | | | |  | |  | | | |  |  | |  | |  | | |  |  |
| Walkers aORs adjusted for limited access to car, distance to workplace, and combines commute with caring responsibilities;  Public transport aORs adjusted for workplace, age group, limited access to car, combines commute with caring responsibilities, and absence of free work car parking | | | | | | | | | | | | | | | | | | | |  |  |
